# EVALUATION OF A SOCIAL WORK AND NURSING INTERPROFESSIONAL TRAINING ON DEMENTIA-FRIENDLY CARE

**DOI:** 10.1093/geroni/igae098.3357

**Published:** 2024-12-31

**Authors:** Leslie Hasche, Pilar Ingle, Michael Talamantes

**Affiliations:** University of Denver, Denver, Colorado, United States; University of Denver, Denver, Colorado, United States; University of Denver, Denver, Colorado, United States

## Abstract

While social work and nursing education offer generalist professional degrees, several initiatives over the last 20 years have infused geriatric education and specialty training to counter the long-standing geriatric workforce shortages. However, these training programs are frequently separated within distinct disciplinary silos with limited opportunities for interprofessional training. Interprofessional education involves a critical pedagogy that increases knowledge about principles, ethics, and values of other professions, while also developing critical thinking skills to promote collaborative working relationships that improve care. The purpose of this study is to evaluate an annual interprofessional cohort training specific to Alzheimer’s Disease and Related Dementias for social work and advanced practice nursing students. From 2019 to 2024, five cohorts (total n= 43, with 24 social work students and 19 advanced practice nursing students) have completed this training. Approximately 30 hours of learning activities supplemented their existing professional degree program education over a 6-month period. These activities included: (1) an immersive training with experiential Second Wind Dreams’ Virtual Dementia Tour, (2) webinar didactic content on dementia-friendly and interprofessional care and interprofessional dyad case consultations, and (3) individual, personalized coaching. Using a pre/post survey adapted from measures of interprofessional collaborative practice and geriatric social work competencies, results demonstrated consistent improvement across each of the domains: values and ethics, roles/responsibilities and communication, assessment and treatment, and teamwork. The positive evaluation findings for increasing interprofessional competencies in dementia-friendly care offer support for expansion to other disciplines, geographical regions, and over sustained time and for strengthening the geriatric workforce.

